# The Effect of Aflatoxin-B_1_ on Red Drum (*Sciaenops ocellatus*) and Assessment of Dietary Supplementation of NovaSil for the Prevention of Aflatoxicosis

**DOI:** 10.3390/toxins5091555

**Published:** 2013-09-16

**Authors:** Katherine E. Zychowski, Aline Rodrigues Hoffmann, Hoai J. Ly, Camilo Pohlenz, Alejandro Buentello, Amelia Romoser, Delbert M. Gatlin, Timothy D. Phillips

**Affiliations:** 1 College of Veterinary Medicine, Texas A&M University, TAMU 4458, College Station, TX 77843, USA; E-Mails: kzychowski@cvm.tamu.edu (K.E.Z.); arodrigues@cvm.tamu.edu (A.R.H.); jly@cvm.tamu.edu (H.J.L.); aromoser@cvm.tamu.edu (A.R.); 2 Department of Wildlife and Fisheries, Texas A&M University, 2258 TAMUS, College Station, TX 77843, USA; E-Mails: cpohlenz@tamu.edu (C.P.); abuentello@schillgen.com (A.B.); d-gatlin@tamu.edu (D.M.G.); 3 Schillinger Genetics, 4401 Westown Parkway, Suite 225, West Des Moines, IA 50266, USA

**Keywords:** red drum, aflatoxin, calcium montmorillonite, NovaSil, histopathology, immune

## Abstract

Aflatoxin B_1_ (AFB_1_) is a potent carcinogen that causes growth stunting, immunosuppression and liver cancer in multiple species. The recent trend of replacing fishmeal with plant-based proteins in fish feed has amplified the AFB_1_ exposure risk in farm-raised fish. NovaSil (NS), a calcium montmorillonite clay, has previously been shown to reduce AFB_1_ bioavailability safely and efficaciously in several mammalian species. This study was designed to: (1) evaluate AFB_1_ impact on cultured red drum, *Sciaenops ocellatus*, over the course of seven weeks; and (2) assess NS supplementation as a strategy to prevent aflatoxicosis. Fish were fed diets containing 0, 0.1, 0.25, 0.5, 1, 2, 3, or 5 ppm AFB_1_. Two additional treatment groups were fed either 5 ppm AFB_1_ + 1% NS or 5 ppm AFB_1_ + 2% NS. Aflatoxin B_1_ negatively impacted red drum weight gain, survival, feed efficiency, serum lysozyme concentration, hepatosomatic index (HSI), whole-body lipid levels, liver histopathological scoring, as well as trypsin inhibition. NovaSil inclusion in AFB_1_-contaminated diets improved weight gain, feed efficiency, serum lysozyme concentration, muscle somatic index, and intraperitoneal fat ratios compared to AFB_1_-treated fish. Although not significant, NS reduced AFB_1_-induced histopathological changes in the liver and decreased Proliferating Cell Nuclear Antigen (PCNA) staining. Importantly, NS supplementation improved overall health of AFB_1_-exposed red drum.

## 1. Introduction

Mycotoxins are toxic metabolites produced by a diverse group of fungi that contaminate agricultural crops prior to harvest or during storage post-harvest [1,2]. Aflatoxin B_1_ (AFB_1_), a mycotoxin produced by *Aspergillus flavus* and *A. parasiticus*, is one of the most potent, naturally-occurring carcinogens known to mankind. Aflatoxin B_1_ exposure causes decreases in weight gain, growth stunting and immunosuppression in animals, while increasing hepatocellular carcinoma incidence [3]. Different species including humans, poultry, swine, and fish all exhibit varying levels of mortality and morbidity upon exposure to AFB_1_ [4,5,6]. However, because the damaging AFB_1_ effects are largely species and dose-specific, additional studies are necessary to determine AFB_1_ susceptibility for at-risk unevaluated species.

As a vital part of the global food industry, aquaculture contributes nearly half of all food of aquatic origin intended for human consumption [7]. Fishmeal, one the most expensive fish feed ingredients, is widely used in the aquaculture industry as the major protein source for farm-raised fish [8]. Menhaden (*Brevoortia* sp.) is a clupeid fish species and the most prevalent form of fishmeal used in North America [9]. Recent studies have been directed toward the development of plant-based alternative protein sources such as soybean, peanut, corn and cottonseed meal [10,11,12,13]. However, incorporation of plant-based ingredients into feed increases the risk for AFB_1_ contamination and subsequent exposure. Aflatoxin B_1_ presence in aquaculture feeds and fish feed ingredients has been well-documented, especially in developing countries [14,15,16].

One strategy to reduce aflatoxin exposure in humans and animals is the use of enterosorption therapy. NovaSil (NS), a calcium montmorillonite clay, binds AFB_1_ in the gastrointestinal tract, thereby reducing overall AFB_1_ bioavailability [17]. With a dioctahedral-layered structure and negatively charged interlayer, NS has high affinity and capacity for AFB_1_ molecules, which exhibit a partial positive charge [18]. Numerous *in vivo* studies have demonstrated the safety and efficacy of this technology [19,20,21], although additional studies are needed to determine the efficacy and proper dosage for farm-raised fish [22].

Red drum, *Sciaenops ocellatus*, is a common recreational and commercial fish native to the Atlantic and Gulf Coast regions of the United States [23]. Red drum is currently farmed in China, Israel, Ecuador and North America [24]. Despite its prevalence and economic importance to the food industry, no studies have evaluated red drum AFB_1_ susceptibility. The study presented here was designed to address two objectives: (1) to evaluate red drum susceptibility to AFB_1_ using a multi-level AFB_1_ challenge incorporated into the feed; and (2) to assess the ability of NS to prevent AFB_1_ toxicity in red drum.

## 2. Results

### 2.1. Growth Parameters

Aflatoxin B_1_ treatment effects, including weight gain (%), survival (%), and feed efficiency, did not result in linear trends, with *R*^2^ values of 0.22, 0.01 and 0.1, respectively. Weight gain of individual treatment means were significantly different, and varied with the 0 ppm AFB_1_ group experiencing the highest weight gain and the 2, 3, and 5 ppm exposure groups exhibiting the least amount of weight gain (Table 1). Likewise, AFB_1_ significantly reduced feed efficiency in a non-linear manner, with the 0 ppm AFB_1_ treatment group demonstrating the highest feed efficiency (0.91) and treated groups ranging from 0.49–0.75. Survival also greatly varied across treatments with 0 ppm AFB_1_ having the highest survival rate.

Among the NS-supplemented treatment groups, only weight gain and feed efficiency were significantly different compared to AFB_1_ controls, with *p*-values of 0.039 and 0.005, respectively. In the case of feed efficiency, 0 ppm AFB_1_ and 5 ppm AFB_1_ were the most significantly different. NovaSil inclusion at both 1% and 2% positively affected weight gain, feed efficiency, and survival after AFB_1_ exposure, although not in a dose-dependent manner.

### 2.2. Immune Response

A summary of immune parameters evaluated for each group is shown in Table 2. The 0.1 ppm AFB_1_-exposed fish exhibited the highest plasma lysozyme values (246 units/mL), while the 5 ppm-exposed fish displayed the lowest levels (45 units/mL). Trypsin inhibition (%) results indicated that 1, 2, 3, and 5 ppm AFB_1_-exposed groups had the lowest percent inhibition and 0.25 ppm AFB_1_ the highest. Additionally, neither the lysozyme nor the trypsin results suggested linearity with an *R*^2^ of 0.3947 and 0.109, respectively. The nitro blue tetrazolium (NBT) test showed no significant differences among any of the AFB_1_-exposed groups.

NovaSil had a significant impact (*p* = 0.021) on the plasma lysozyme concentration with 5 ppm AFB_1_ + 2% NS outperforming all other treatments. NovaSil did not significantly alter levels of NBT or trypsin inhibition.

### 2.3. Somatic Indexes

Somatic indexes for spleen, MSI and IPF did not vary within the AFB_1_-treated groups; however, HSI varied slightly between treatments. The highest HSI levels were recorded in the 0.1 AFB_1_-treated group, while the 2 ppm and 5 ppm exposure groups exhibited the lowest values (Table 3). A linear trend was not present in any of the groups.

In the NS-supplemented groups, muscle and IPF levels recovered to control levels in the treatment group administered 2% NS. Likewise, the means of main effect data indicate that NS inclusion at either 0% and 1% was statistically different than 2%.

**Table 1:** Growth performance of red drum fed different concentrations of Aflatoxin B_1_ (AFB_1_) ^1^ and AFB_1_ + NovaSil (NS) ^2,3,4^.

Variable	Weight gain ^5^ (%)	Survival (%)	Feed efficiency	Variable		Weight gain (%)	Survival (%)	Feed efficiency
AFB_1_ (ppm)	Individual treatment means			AFB_1_ (ppm)	NS (%)	Individual treatment means		
0	332 ^a^	80.0 ^a^	0.91 ^a^	0	0	332 ^ab^	80.0	0.91 ^a^
0.1	223 ^bc^	46.6 ^b^	0.62 ^bc^	5	0	188 ^c^	55.5	0.62 ^c^
0.25	224 ^bc^	55.5 ^b^	0.65 ^bc^	5	1	339 ^a^	73.3	0.82 ^ab^
0.5	254 ^ab^	60.0 ^ab^	0.75 ^ab^	5	2	218 ^bc^	57.7	0.71 ^bc^
1	212 ^bc^	60.0 ^ab^	0.73 ^abc^	*p*-value		0.039	0.261	0.005
2	136 ^c^	60.0 ^ab^	0.49 ^c^	Pooled Std. Error		7.047	1.801	0.008
3	183 ^bc^	62.2 ^ab^	0.67 ^bc^	**AFB_1_ (ppm)**	**NS (%)**	**Means of main effect**		
5	188 ^bc^	55.5 ^b^	0.62 ^bc^	0		332	80.0	0.91 ^a^
*R^2^*	0.229	0.010	0.100	5		249	62.2	0.72 ^b^
*p*-value	0.005	0.132	0.030		0	260 ^a^	67.7	0.77
Pooled Std. Error	5.189	1.309	0.013		1	339 ^ab^	73.3	0.82
					2	218 ^b^	57.7	0.71
						**ANOVA: *p*-values**		
				**AFB_1_**		0.083	0.138	0.003
				**NS**		0.043	0.387	0.029

^1^ Aflatoxin B_1_; ^2^ NovaSil; ^3^ Values are means of three replicate groups of fish (*n* = 3); ^4^ Values in a column that do not have the same superscript are significantly different according to Duncan’s multiple range test (*p* < 0.05); ^5^ Initial average weight was 2.1 ± 0.1 g/fish.

**Table 2:** Immune parameters of red drum ^1^.

Variable	Serum lysozyme (units/mL)	NBT (mg/mL blood) ^2^	Trypsin inhibition (%)	Variable		Serum Lysozyme (units/mL)	NBT (mg/mL blood) ^2^	Trypsin inhibition (%)
AFB_1_ (ppm)	Individual treatment means			AFB_1_ (ppm)	NS (%)	Individual treatment means		
0	165 ^ab^	3.52	83.6 ^ab^	0	0	165 ^ab^	3.52	83.6
0.1	246 ^a^	3.35	82.4 ^b^	5	0	45 ^c^	3.07	81.9
0.25	131 ^bcd^	2.54	86.3 ^a^	5	1	76b ^c^	3.32	81.3
0.5	155 ^abc^	3.30	83.2 ^b^	5	2	185 ^a^	3.21	79.4
1	106 ^bcd^	1.78	81.9 ^b^	*p*-Value		0.024	0.944	0.577
2	82 ^bcd^	3.05	80.5 ^b^	Pooled Std. Error		5.550	0.104	0.395
3	63 ^cd^	2.21	82.7 ^b^	**AFB_1_ (ppm)**	**NS (%)**	**Means of main effect**		
5	45 ^d^	3.07	81.9 ^b^	0		165	3.52	83.6
*R* ^2^	0.394	0.015	0.109	5		102	3.20	80.9
*p*-Value	0.004	0.250	0.038		0	105	3.30	82.7
Pooled Std. Error	5.705	0.102	0.192		1	76	3.32	81.3
					2	185	3.21	79.4
						**ANOVA: *p*-Values**		
				**AFB_1_**		0.018	0.622	0.291
				**NS**		0.021	0.948	0.674

^1^ Values in a column that do not have the same superscript letters are significantly different according to Duncan’s multiple range test (*p* < 0.05); ^2^ Values are means of determinations on two fish from each of three replicate groups (6 fish/treatment, *n* = 6).

**Table 3:** Somatic indices of red drum fed different concentrations of AFB_1_^1^ and AFB_1_ + NS ^2,3,4^.

Variable	Spleen	MSI ^5^	HSI ^6^	IPF ^7^	Variable		Spleen	MSI ^5^	HSI ^6^	IPF ^7^
AFB_1_ (ppm)	Individual treatment means				AFB_1_ (ppm)	NS (%)	Individual treatment means			
0	0.04	28.94	1.67 ^abc^	0.32	0	0	0.04	28.94 ^a^	1.67	0.32 ^a^
0.1	0.04	27.42	1.98 ^a^	0.11	5	0	0.04	26.06 ^b^	0.88	0.01 ^b^
0.25	0.04	26.18	1.79 ^ab^	0.20	5	1	0.09	28.33 ^ab^	0.82	0.10 ^b^
0.5	0.05	27.87	1.20 ^abc^	0.26	5	2	0.20	29.70 ^a^	1.56	0.46 ^a^
1	0.03	27.79	1.15 ^abc^	0.07	*p*-Value		0.528	0.031	0.292	0.003
2	0.18	26.25	0.72 ^c^	0.18	Pooled Std. Error		0.015	0.135	0.070	0.012
3	0.05	25.72	0.94 ^abc^	0.06	**AFB_1_ (ppm)**	**NS (%)**	**Means of main effect**			
5	0.04	26.06	0.88 ^bc^	0.01	0		0.04	28.94	1.67	0.32
*R^2^*	0.004	0.141	0.267	0.152	5		0.11	28.03	1.09	0.19
*p*-Value	0.503	0.417	0.091	0.466		0	0.04	27.50	1.27	0.17 ^a^
Pooled Std. Error	0.015	0.315	0.091	0.03		1	0.09	28.33	0.82	0.10 ^a^
						2	0.20	29.70	1.56	0.46 ^b^
							**ANOVA: *p*-Values**			
					**AFB_1_**		0.494	0.298	0.205	0.112
					**NS**		0.429	0.019	0.332	0.002

^1^ Aflatoxin B1; ^2^ NovaSil; ^3^ Values in a column that do not have the same superscript letters are significantly different according to Duncan’s multiple range test (*p* < 0.05); ^4^ Values are means of determinations on two fish from each of three replicate groups (6 fish/treatment, *n* = 6); ^5^ Muscle somatic index; ^6^ Hepatosomatic index; ^7^ Intraperitoneal fat.

### 2.4. Proximate Composition

No linear trends were present in the AFB_1_-treated groups (Table 4). Percent lipid composition was highest in the 0 ppm AFB_1_ group and the lowest at 2 ppm AFB_1_, but varied among other treatments. There were some variations in ash values as well; however, these results were not linearly correlated. Inclusion of NS in the diets did not exhibit any statistically significant changes in whole-body proximate composition.

**Table 4:** Proximate composition of red drum (fresh-weight basis) ^1,2^.

Variable	% Lipid	% Protein	% Moisture	% Ash	Variable		% Lipid	% Protein	% Moisture	% Ash
AFB_1_ (ppm)	Individual treatment means				AFB_1_ (ppm)	NS (%)	Individual treatment means			
0	2.70 ^a^	76.01	78.38	16.38 ^ab^	0	0	2.21	76.01	78.30	3.54
0.1	2.37 ^ab^	74.45	79.34	17.56 ^a^	5	0	1.98	76.52	79.29	3.73
0.25	1.97 ^bcd^	70.06	79.67	13.64 ^b^	5	1	2.20	73.92	76.91	4.28
0.5	2.42 ^ab^	74.33	77.69	16.72 ^ab^	5	2	2.19	72.93	79.04	4.35
1	2.17 ^abc^	71.64	78.71	18.04 ^a^	*p*-Value		0.510	0.723	0.173	0.629
2	1.45 ^d^	74.20	84.55	19.43 ^a^	Pooled Std. Error		0.022	0.488	0.140	0.098
3	1.77 ^cd^	71.64	80.60	17.22 ^a^	**AFB_1_ (ppm)**	**NS (%)**	**Means of main effect**			
5	1.98 ^bcd^	76.52	79.29	18.02 ^a^	0		2.21	76.01	78.38	3.54
*R^2^*	0.211	0.021	0.024	0.109	5		2.12	74.46	78.41	4.12
*p*-Value	0.002	0.476	0.452	0.038		0	2.10	76.20	78.83	3.64
Pooled Std. Error	0.033	0.441	0.402	1.728		1	2.20	73.90	76.91	4.28
						2	2.19	72.90	79.04	4.35
							**ANOVA: *p*-Values**			
					**AFB_1_**		0.534	0.611	0.964	0.357
					**NS**		0.394	0.604	0.095	0.663

^1^ Values are means of determinations on three fish from each of the three replicates (*n* =3). ^2^ Values in a column that do not have the same superscript letters are significantly different according to Duncan’s multiple range test (*p* < 0.05).

### 2.5. Histopathological Response and Immunohistochemistry

**Figure 1:**
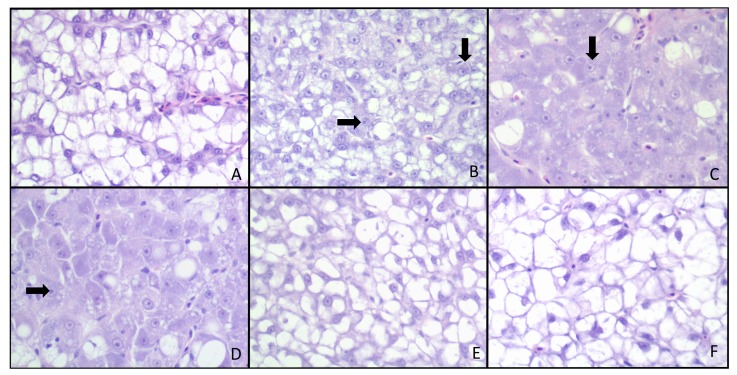
Liver histopathology in AFB_1_-exposed red drum. Liver sections were stained with hematoxylin and eosin. Treatments were as follows: (**A**) 0 ppm AFB_1_ (**B**) 1 ppm AFB_1_ (**C**) 3 ppm (**D**) 5 ppm AFB_1_ (**E**) AFB_1_ + 1% NS and (**F**) 5 ppm AFB_1_ + 2% NS. Marked pleomorphism, megalokaryosis with prominent nucleoli (arrows) and loss of hepatocellular cytoplasmic macrovacuolation was observed in the treatment groups that received large amounts of aflatoxin (**B**,**C**,**D**). Although not significant, inclusion of NS resulted in decreased histopathological scores attributable to increased cytoplasmic vacuolation and reduced cellular pleomorphism.

**Figure 2:**
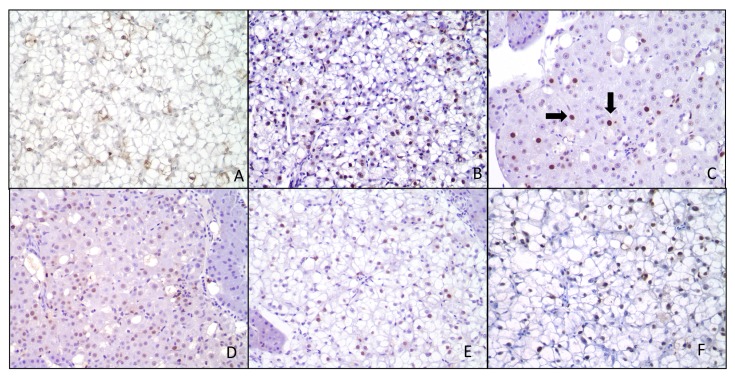
Proliferating Cell Nuclear Antigen (PCNA) positive cells in red drum hepatocytes. Liver sections were stained with PCNA (arrows) and hematoxylin counterstain. Treatments were as follows: (**A**) 0 ppm AFB_1_ (**B**) 1 ppm AFB_1_ (**C**) 3 ppm AFB_1_ (**D**) 5 ppm AFB_1_ (**E**) 5 ppm AFB_1_ + 1% NS (**F**) 5 ppm AFB_1_ + 2% NS. Although not significant, inclusion of NS resulted in a decrease of PCNA-positive hepatocytes. Reduction in cell proliferation suggests that NS afforded some protection from AFB_1_ toxicity and cellular proliferation.

Significant histological changes were observed between treatments (Table 5), with 3 and 5 ppm AFB_1_ eliciting the most severe hepatic alterations. Although some samples revealed significant hepatic lesions in groups treated with 5 ppm AFB_1_ + 1% or 2% NS, the findings in these fish were considered mild when compared to the 5 ppm AFB_1_ without NS. There were no significant differences in Proliferating Cell Nuclear Antigen (PCNA) values among all treatments, nor did PCNA staining exhibit a positive linear correlation. Histological changes, characterized by restoration of hepatocellular macrovacuolation and reduced megalocytosis and karyomegaly, were noted with the addition of NS in the diet; however, these results were not statistically significant (Figure 1). A decrease in PCNA staining as compared to the 5 ppm inclusion level was noted, but also did not achieve significant levels with 1% or 2% NS inclusion in the diet (Figure 2).

**Table 5:** Histopathology and immunohistochemistry.

Variable	Histology Score ^1^	PCNA	Variable		Histology Score	PCNA
AFB_1_ (ppm)	Individual treatment means		AFB_1_ (ppm)	NS (%)	Individual treatment means	
0	5.25 ^a^	6.27	0	0	13.16 ^ab^	6.27
0.1	10.67 ^a^	8.59	5	0	19.00 ^b^	10.49
0.25	17.33 ^ab^	9.35	5	1	9.16 ^a^	9.11
0.5	30.16 ^c^	11.34	5	2	7.66 ^a^	9.72
1	25.83 ^bc^	9.52	*p*-Value		0.0925	0.7542
2	31.83 ^c^	9.06	Pooled Std. Error		0.838	0.836
3	37.00 ^c^	10.30	**AFB_1_ (ppm)**	**NS (%)**	**Means of main effect**	
5	37.00 ^c^	10.49	0		13.16	6.27
*R* ^2^	0.2353	0.0204	5		11.94	9.78
*p*-Value	0.0001	0.5059		0	16.08	8.38
Pooled Std. Error	1.130	0.815		1	9.16	9.11
				2	7.66	9.72
					**ANOVA: *p*-Values**	
			**AFB_1_**		0.7248	0.3251
			**NS**		0.0491	0.9454

^1^ Values in a column that do not have the same superscript letters are significantly different according to Duncan’s multiple; range test (*p* < 0.05).

## 3. Discussion

Aflatoxin B_1_ displayed a significant effect across multiple treatment levels. The survival rate for the basal diet group (0 ppm AFB_1_) was similar to control survival results reported in other red drum studies [25,26], although survival was negatively affected by AFB_1_ presence. Likewise, the impact on feed efficiency and weight gain found in this study has been similarly documented in other AFB_1_-exposure publications, including research analyzing the effects of aflatoxins on several different farmed aquatic species [27,28,29,30,31]. The majority of AFB_1_-sensitive ichthyoids are cold-water species and our findings suggest that red drum may be one of the first identified AFB_1_-sensitive warm-water species. However additional studies are necessary to determine the specific metabolic mechanisms responsible for this sensitivity.

The published aquaculture literature indicates that incremental increases in AFB_1_ exposure do not typically result in dose-dependent, linear responses [32,33,34]. Herein, analysis of growth performance factors indicated that some of the most significant AFB_1_ effects were present at the lowest level of AFB_1_-exposure (0.1 ppm) for feed efficiency, survival, and weight gain. Hormetic responses for growth and immunological parameters have been observed in several species [35]. Hormesis is defined as a biphasic response to a xenobiotic, characterized by a low-dose stimulatory effect and high-dose inhibitory or toxic effect in which a U-shaped or J-shaped model is apparent [36]. Instances of AFB_1_-associated hormesis have also been documented in multiple species [32,37,38]. Several measured parameters in the current study suggest that AFB_1_-exposed red drum exhibited an “inverted U-shaped” immunological hormetic response to AFB_1_ as suggested by plasma lysozyme at the 0.1 ppm level and trypsin inhibition at the 0.25 ppm level. Additionally, HSI results indicated a similar increase at the 0.1 ppm level followed by subsequent decreases at higher AFB_1_ levels.

Several studies have indicated that PCNA is a suitable marker for cellular proliferation in fish [39,40] as well as other species [41,42]. However, our study did not indicate any significant differences in PCNA staining among the treatments. It is possible that the levels of AFB_1_ used in this study were not capable of inducing significant cellular proliferation as observed with other species. While there was a slight increase in PCNA with the presence of AFB_1_, there was a decrease in HSI. The increase in PCNA is due to liver damage and mitotic activity from AFB_1_-exposure, while the overall decrease in HSI is likely attributed to the loss of vacuolation and fat in the liver. Histological evaluation indicated liver changes characterized by anisokaryosis, megalocytosis and karyomegaly in AFB_1_-exposed red drum, which have been noted in a series of AFB_1_ studies with other fish species [43,44,45]. Hepatocellular lipid deposition, a well-documented classical sign of aflatoxicosis in fish [46,47], was present in red drum exposed to AFB_1_. However, red drum kept in captivity typically display fatty deposition and hepatocellular macrovacuolation [48], which should be taken into consideration for accurate red drum liver evaluation. The hepatocellular vacuolation seen in control livers was markedly reduced, as anisocytosis and karyomegaly increased, especially in fish exposed to higher levels of aflatoxin. Interestingly, hepatocellular vacuolation and liver fat were restored in fish treated with NS. Ideally, further red drum AFB_1_ studies should pair liver histological evaluation with other molecular markers to confirm liver damage, such as inducible nitric oxide synthase ([49] or γ-glutamyl transpeptidase [50,51]. Additionally, because feed efficiency, IPF and liver fat decreased with AFB_1_ exposure, it is possible that there was increased energy expenditure in these fish because less food was utilized. However, more research is needed to determine the exact mechanism of fat loss in AFB_1_-exposed red drum.

In this study, NS supplementation in the diets of AFB_1_-exposed fish resulted in a protective effect, which was evident by the significant improvement in many of the tested parameters. Other studies have reported that a 2% inclusion level of bentonite, a common clay containing montmorillonite, in trout feed reduced toxic AFB_1_ effects [52]. Yet other studies suggest that a 0.5% inclusion level was sufficient to protect tilapia from 1.5 ppm AFB_1_ [45]. Bentonites have been added into fish feed at concentrations up to 10% with no alteration in whole-body proximate composition [53]. Discrepancies in the aquaculture literature concerning the proper inclusion level of clay-based binders indicate a need to establish a clay dosing regimen for fish at risk for AFB_1_ exposure.

## 4. Materials and Methods

### 4.1. Experimental Diets

The control basal diet was composed of 400 g protein kg^−1^ and 110 g lipid kg^−1^, containing an estimated 3.5 kcal digestible energy kg^−1^ (Table 6) and fulfilling all documented nutrient requirements of red drum [54]. Aflatoxin B_1_ (Sigma-Aldrich, St. Louis, MO, USA) was incorporated into the diet by first dissolving the AFB_1_ in chloroform and subsequently adding it to Celufil, a non-nutritive bulking agent (USB Corporation, Cleveland, OH, USA). The chloroform was evaporated to dryness from the mixture in a dark room under a fume hood, leaving the Celufil amended with AFB_1_. A V-mixer was used to blend all dry ingredients, with the exception of the AFB_1_-spiked Celufil, for 20 min. The dry ingredients were then mixed with the AFB_1_-spiked Celufil in a Hobart mixer until homogeneity was achieved. The oil component and 700 mL of H_2_O were further added to the dry ingredients and mixed for 1 h. Aflatoxin-free Celufil was incorporated into the basal diet for comparison. The moist feed was cold-pelleted through a 3-mm die on a meat grinder attachment and dried in a dark room for 24 h. Diets were subsequently bagged and stored at −20 °C until needed. The ten diets contained the following: 0 ppm AFB_1_ (*i.e.*, 0 ppm AFB_1_ + 0% NS), 0.1 ppm AFB_1_, 0.25 ppm AFB_1_, 0.5 ppm AFB_1_, 1 ppm AFB_1_, 2 ppm AFB_1_, 3 ppm AFB_1_, 5 ppm AFB_1_, 5 ppm AFB_1_ + 1% NS and 5 ppm AFB_1_ + 2% NS. A NS control group was not included since its safety was previously evaluated over the course of 10 weeks in a similar warm-water species [22].

### 4.2. Fish Stock and Culture Conditions

Fingerling red drum were transported from the Texas Parks and Wildlife hatchery located at Lake Jackson, TX to the Texas A&M Aquacultural Research and Teaching Facility. Fish were stocked and conditioned in round tanks with a commercial diet (Rangen, Inc., Angelton, TX, USA) for 2 weeks, then transferred to aquaria and conditioned for 1 week on the basal diet. A closed, re-circulating system was composed of 110 L aquaria with water flowing at 1 L/min. Biofiltration was used to maintain ammonia, nitrate and nitrite concentrations at non-toxic levels. Salinity was maintained at 7 ppt with artificial salts and water temperature was kept constant at 37 ± 2 °C by controlling air temperature in the wet laboratory. Supplemental aeration provided an adequate dissolved oxygen level of at least 80% air saturation. A 12:12 h light:dark cycle was maintained throughout the conditioning and trial period and water quality was monitored on a daily basis. Fifteen fish (2.1 ± 0.1 g) were stocked in each aquarium. The 10 dietary treatments were randomly assigned to triplicate aquaria, requiring a total of 30 tanks. Fish were fed a morning and afternoon ration over the course of 7 weeks. The diets were fed to fish beginning at a rate of 6% of the initial body weight and tapered to 3% over the span of the trial to prevent overfeeding and to approach apparent satiation. The system was monitored for mortalities and any deceased fish were immediately removed and evaluated for cause of death. With the exception of weight gain and survival, which were monitored on a weekly and daily basis, respectively, all other parameters were evaluated at the end of 7 weeks.

**Table 6:** Ingredient and proximate composition of experimental diets (g/100 g of dry weight).

Level of AFB_1_ (ppm)	0	0.1	0.25	0.5	1	2	3	5	5	5
Level of NS (%)	0	0	0	0	0	0	0	0	1	2
Menhaden Meal ^a^	34.9	34.9	34.9	34.9	34.9	34.9	34.9	34.9	34.9	34.9
Soybean Meal ^b^	27.3	27.3	27.3	27.3	27.3	27.3	27.3	27.3	27.3	27.3
Dextrinized Starch ^c^	16.0	16.5	16.5	16.5	16.5	16.5	16.5	16.5	16.5	16.5
Menhaden Oil ^a^	5.6	5.6	5.6	5.6	5.6	5.6	5.6	5.6	5.6	5.6
Vitamin Premix ^d^	3.0	3.0	3.0	3.0	3.0	3.0	3.0	3.0	3.0	3.0
Mineral Premix ^c^	4.0	4.0	4.0	4.0	4.0	4.0	4.0	4.0	4.0	4.0
CMC ^c^	2.0	2.0	2.0	2.0	2.0	2.0	2.0	2.0	2.0	2.0
Glycine ^e^	1.0	1.0	1.0	1.0	1.0	1.0	1.0	1.0	1.0	1.0
Lysine ^e^	0.0	0.0	0.0	0.0	0.0	0.0	0.0	0.0	0.0	0.0
NS ^f^	0.0	0.0	0.0	0.0	0.0	0.0	0.0	0.0	1.1	2.3
AFB_1_-spiked Celufil ^g^	0.0	0.2	0.7	1.6	4.5	0.5	0.8	1.6	1.6	1.6
Celufil ^e^	5.5	5.3	4.8	3.9	1.0	5.0	4.7	3.9	2.8	1.7
**Proximate Composition (% dry matter)**										
Protein	36.2	35.8	35.7	35.2	35.6	35.5	36.1	35.6	35.2	35.5
Lipid	9.5	9.3	10.3	10.4	10.7	10.6	10.6	10.7	10.7	11.1
Dry Matter	94.5	94.7	94.9	93.6	94.3	94.8	95.2	95.4	95.4	95.0
Ash	11.1	10.9	10.9	11.3	11.1	10.9	10.9	11.1	11.8	12.9

^a^ Special Select, Omega Protein, Houston, TX, USA; ^b^ De-hulled, roasted/cooked and solvent extracted, Producers Cooperative Association, Bryan, TX; ^c^ MP Biomedicals LLC, Solon, OH; ^d^ Contains (as g kg^−1^): Ca(C_6_H_10_O_6_)·5H_2_O, 348.49; Ca(H_2_PO_4_)·2H_2_O, 136.0; FeSO_4_·7H_2_O, 5.0; MgSO_4_·7H_2_O, 132.0; K_2_HPO_4_, 240.0; NaH_2_PO_4_·H_2_O, 88.0; NaCl, 45.0; AlCl_3_ 6H_2_O, 0.15; KI, 0.15; CuSO_4_·5H_2_O, 0.5; MnSO_4_·H_2_O, 0.7; CoCl_2_·6H_2_O, 1.0; ZnSO_4_·7H_2_O, 3.0; Na_2_SeO_3_, 0.011; ^e^ USB Corporation, Cleveland, OH; ^f^ Englehard Corporation, Jackson, MS; ^g^ Sigma-Aldrich, St. Louis, MO, USA.

### 4.3. Fish Growth and Health Responses

Weight gain (% of initial weight), feed efficiency (g weight gain/g dry diet fed), and survival rate (% per treatment group) were calculated at the end of the trial. Two fish were sampled from each aquaria and homogenized together using a blender. Whole-body analysis was performed by evaluating moisture, ash, protein and lipid content according to previously established procedures [55]. Somatic indexes including spleen, liver (HSI), intraperitoneal (IPF) fat and muscle (MSI) were averaged based on 2 fish per aquaria (*n* = 6). Each somatic index was calculated as follows: (organ weight/body weight) × 100. Only the dextral side of each fish was filleted, weighed, and then doubled to obtain MSI.

### 4.4. Immunological Responses

Immunological parameters were evaluated including plasma lysozyme of white blood cell origin, neutrophil oxidative radical production in whole blood, and % trypsin inhibition. Two fish were randomly selected and bled from each tank, then pooled according to treatment (6 fish per treatment). A total of approximately 1–2 mL of blood was collected per treatment group using heparinized syringes. Plasma lysozyme was analyzed by employing a turbidimetric method [56,57]. Blood neutrophil oxidative radical production was measured utilizing a nitro blue tetrazolium (NBT) assay [56,58]. Plasma was also used to determine % trypsin inhibition according to a previously established method [59].

### 4.5. Histological Response

Livers were dissected from two fish per tank, or six per treatment. Immediately after dissection, livers were fixed in 10% formalin overnight. Livers were subsequently rinsed with 70% ethanol solution and transferred to vials containing 10 mL fresh 70% ethanol. Samples were processed and paraffin embedded within 48 h for routine histopathology at the Texas A&M Veterinary Pathobiology Histology Laboratory (College Station, TX, USA). Samples were sectioned at a thickness of 5 µm and stained with hematoxylin and eosin (H&E). Lesions were blindly examined and scored according to the criteria listed in Table 7.

**Table 7:** Histological evaluation criteria.

Score	Evaluation	Description
0	Normal	Intracytoplasmic vacuolation, mostly macrovacuolar with one of the control livers also having micro and macrovesiculation. Nuclei are small and pushed to the periphery with small nucleoli.
1+	Minimal	Scattered increase in nuclear size and mostly inconspicuous nucleoli.
2+	Mild	Mild hypertrophy and pleomorphism with slightly prominent nuclei and more evident nucleoli. Some loss of intracytoplasmic macrovacuoles, and formation of microvacuoles.
3+	Moderate	Moderate cellular pleomorphism, with anisocytosis, anisokaryosis, megalocytosis and megalokaryosis. Sparse intracytoplasmic vacuoles.
4+	Marked	Diffuse loss of cytoplasmic vacuolation, mostly solid cytoplasm. Marked pleomorphism, anisocytosis, anisokaryosis, megalocytosis and megalokaryosis.

### 4.6. Immunohistochemistry

Immunohistochemistry for proliferating cell nuclear antigen (PCNA) was performed on deparaffinized sections of liver mounted on positively charged, silanized slides using an automated staining system for immunohistochemistry (Lab Vision Autostainer 360, Runcom, Cheshire, UK). Briefly, slides were placed in a heated chamber with DIVA decloaking solution (Biocare Medical LLC., Concord, CA, USA) and heated to 121 °C for antigen retrieval. The slides were incubated with a 1:200 dilution of PCNA (Fisher Scientific, Walther, MA, USA) for 20 min followed by a secondary antibody, ImmpRESS (Vector Scientific, Burlingame, CA, USA) for 30 min. The primary antibody was omitted on negative control tissues. Slides were then stained with DAB Quanto (Vector Scientific, Burlingame, CA, USA) for 5 min, followed by counterstaining with hematoxylin (Biocare Medical LLC., Concord, CA, USA) for 1.5 min. Slides were further dehydrated and mounted. Negative and positive control tissues were stained together with all fish livers. Canine and mouse small intestine, bronchial epithelium and tonsils were used as positive control tissues. All photographs were taken at 400× magnification. Stained nuclei were counted, averaged and evaluated for each treatment using CellProfiler software [60]. The percentage of PCNA positive cells ((positive/total nuclei) ×100) was calculated based on 4 fields/fish × 6 fish/treatment (24 fields/treatment).

### 4.7. Statistical Analysis

All statistics were computed using Statistical Analysis System (SAS) version 9.2 (SAS Institute, Cary, NC, USA). Data from groups exposed to 0–5 ppm AFB_1_ were subject to a general linear model regression, while 0 ppm AFB_1_, 5 ppm AFB_1_, 5 ppm AFB_1_ + 1% NS and 5 ppm AFB_1_ + 2% NS group data were subject to an incomplete factorial ANOVA for all parameters except histopathological scoring. Histopathological scores were first subject to Aligned Rank Transformation [61] and then further analyzed using a general linear model regression or incomplete factorial ANOVA. All differences among treatment means were determined using Duncan’s multiple range test. Treatment differences were considered significant at *p* < 0.05.

## 5. Conclusions

These findings indicate that red drum are susceptible to AFB_1_ in levels as low as 0.1 ppm. Other unevaluated species should be tested for AFB_1_ susceptibility, especially warm-water species raised in tropical and subtropical environments where the mycotoxin contamination risk is high. NovaSil supplementation at levels between 1%–2% may be used in fish feed safely to effectively reduce AFB_1_ toxicity. Therefore, this technology could be used by the aquaculture industry as a strategy to reduce aflatoxin-related morbidity and mortality in fish.
